# Pathology of Red Blood Cells in Patients with SARS-CoV-2

**DOI:** 10.3390/biomedicines13010191

**Published:** 2025-01-14

**Authors:** Sona Hakobyan, Lina Hakobyan, Liana Abroyan, Aida Avetisyan, Hranush Avagyan, Nane Bayramyan, Lyudmila Niazyan, Mher Davidyants, Knarik Sargsyan, Tehmine Ghalechyan, Anna Semerjyan, Elena Karalova, Zaven Karalyan

**Affiliations:** 1 Laboratory of Cell Biology and Virology, Institute of Molecular Biology of NAS RA, Yerevan 0014, Armeniaa.avetis@mail.ru (A.A.); a.avagian@yahoo.com (H.A.); zkaralyan@yahoo.com (Z.K.); 2 Experimental Laboratory, Yerevan State Medical University, Yerevan 0093, Armenia; 3 National Center of Infectious Diseases, Ministry of Health, RA, Yerevan 8424, Armeniadavidyants@gmail.com (M.D.); t.ghalechyan88@gmail.com (T.G.); 4 Department of Medical Biology, Yerevan State Medical University, Yerevan 0025, Armenia

**Keywords:** COVID-19, red blood cells, hemoglobin adsorption, erythroblastosis, microspectrophotometry

## Abstract

** Background:** Severe acute respiratory syndrome coronavirus 2 (SARS-CoV-2) infection has been associated with various hematological disorders. Understanding the pathology of erythrocytes (red blood cells) in coronavirus infection may provide insights into disease severity and progression. **Objective:** To review and analyze the general pathology of erythrocytes in patients infected with SARS-CoV-2, focusing on clinical and laboratory findings across different severity groups. **Methods:** Patients were classified into four groups based on clinical criteria: Group 1: Regular group (fever, respiratory symptoms, and radiographic evidence of pneumonia). Group 2: Severe group (shortness of breath >30 breaths/min, peripheral blood oxygen saturation <92% at rest, extensive pneumonia, respiratory failure requiring mechanical ventilation, and/or organ failure necessitating intensive care). Group 3: Low saturation group (peripheral blood oxygen saturation <85% at rest). Group 4: Erythroblastosis group (erythroblast count >0.5% among total nucleated blood cells). Clinical laboratory investigations included major routine studies and scanning microspectrophotometry to measure hemoglobin (Hb) spectra in unstained erythrocytes. **Results:** Erythroblasts were detected in approximately 30% of SARS-CoV-2 patients, predominantly in the severe group. Serum ferritin, C-reactive protein (CRP), and anisocytosis were strongly correlated with disease severity. Microspectrophotometric studies revealed significant changes in hemoglobin adsorption spectra, with an increase in Hb absorbance at 420 nm in severe cases compared to normal controls. **Conclusions:** Elevated serum ferritin, CRP levels, anisocytosis, and altered hemoglobin absorption at 420 nm wavelength are associated with adverse outcomes in SARS-CoV-2 infection. These findings highlight the potential utility of hematological parameters as markers for disease severity and prognosis in viral infections.

## 1. Introduction

There are several subfamilies of coronaviruses. Seven coronaviruses have been described to cause respiratory diseases in humans. Four of these are common human coronaviruses, and one of the seven is severe acute respiratory syndrome coronavirus 2 (SARS-CoV-2). SARS-CoV-2 belongs to the subfamily Coronavirinae. Other coronavirus species capable of causing severe human diseases include SARS-CoV and MERS-CoV. The coronaviruses HKU1, HCoV-NL63, HCoV-OC43 and HCoV-229E are associated with mild symptoms in humans [1,2].

Coronavirus disease 19 (COVID-19) is an infectious-inflammatory disease that primarily affects the lungs. The severe course of the disease is associated with multi-organ pathology with different routes of injury. Although erythrocyte pathology is less commonly mentioned, several articles have suggested that SARS-CoV-2 infection may cause hematological disorders [3]. Authors usually point to differences in hematological manifestations between severe and non-severe patients. Hemoglobinopathy, hypoxia, and cellular iron overload may play a role in SARS-CoV-2 pathology. Two potential pathophysiological mechanisms have been suggested in the scientific literature: a) interaction of SARS-CoV-2 with the hemoglobin molecule through CD147, CD26, and other receptors located on erythrocytes and/or blood cell precursors; b) hepcidin-mimetic action of a viral spike protein inducing ferroportin blockade [3].

Serum ferritin is known as an iron storage protein and is commonly measured as an indicator of iron status. It is also a prominent marker of inflammation, with serum ferritin levels rising significantly in response to inflammation and other pathologies. Serum ferritin levels also correlated with disease severity in SARS-CoV-2 patients, but the mechanisms for the association between hyperferritinemia and disease severity in SARS-CoV-2 patients remained unclear [3].

Normally, erythroblasts should be completely absent in adult blood and are usually observed in almost all forms of severe anemia (except aplastic). Several investigations have reported erythroblastosis in SARS-CoV-2 patients [4,5,6]. Ferritin has been shown to be frequently located on the plasma membrane of erythroblasts [7]. Thus, the purpose of this study is to investigate/examine the general pathophysiology of red blood cells in coronavirus infection, especially to look into the association/relationship between serum ferritin levels with erythroblastosis and hemoglobin abnormalities in patients with SARS-CoV-2.

## 2. Methods

### 2.1. Virus

Within the experiments, a delta variant of SARS-CoV-2 was used [8,9].

### 2.2. Patients

This study was approved by the Institutional Review Board/Independent Ethics Committee of the Institute of Molecular Biology of the National Academy of Sciences, Yerevan, Armenia. The study was approved by the Institutional Review Board of the Institute of Molecular Biology NAS RA IRB00004079.

We performed quantitative polymerase chain reaction (qRT-PCR) (both using extracted RNA and direct samples (initial swab samples)) targeting the N gene and the ORF1ab gene in the conserved region of the SARS-CoV-2 genome [10]. The current study included 74 patients with SARS-CoV-2 who were treated at the National Centre for Infectious Diseases, Ministry of Health of Armenia. The study was conducted from May to September 2020.

Patient inclusion criteria are presented below:

- Patients diagnosed with SARS-CoV-2 within positive SARS-CoV-2 qRT-PCR.
- Residents of the National Centre of Infectious Diseases, Ministry of Health, Republic of Armenia.
- Age of patients 18 years and older.
- Negative pregnancy test for females.
- Informed consent is required from the patient or his/her representative to participate in the study.
- The patient should be able to comply with all the requirements of the clinical trial (including home follow-up during isolation).

Patient exclusion criteria are presented below.

- Known history of allergy.
- Positive IgG to SARS-CoV-2 was obtained prior to testing.
- Any of the following comorbidities (or any other condition that may interfere with the study): Immunosuppression. Chronic obstructive pulmonary disease. Obesity. Acute or chronic renal insufficiency. History of severe coronary disease. History of cerebrovascular disease. Current neoplasm.

Computed tomography (CT) of the lungs was performed in all patients.

### 2.3. Clinical Criteria

The classification of the patients in the study was in accordance with Yuan [11]:

I. Regular group (fever, respiratory symptoms, and radiographic evidence of pneumonia),

II. Severe group patients with shortness of breath (more than 30 breaths per minute), peripheral blood oxygen saturation less than 92% at rest, pneumonia involving more than 50% of the tissues, and/or respiratory failure requiring mechanical ventilation support and/or organ failure requiring intensive care.

III. The low saturation group with peripheral blood oxygen saturation less than 85% at rest (before mechanical ventilation support).

IV. Erythroblastosis group with erythroblast count greater than 0.5% of total nucleated blood cells.

### 2.4. Laboratory Measurements

Clinical laboratory investigations included a complete blood count and the determination of ferritin, C-reactive protein (CRP), and lactose dehydrogenase (LDH) in patients’ sera.

Blood samples were analyzed using commercially available ELISA kits that are normally used in the hospital’s clinical practice [12,13].

### 2.5. Blood Smears, Giemsa Staining, and Nucleated Blood Cells Analysis

Blood smears were prepared from fresh blood using routine methods. For nucleated blood cell analysis, slides were fixed in pure methanol and stained with Giemsa-modified solution (azure B/azure II, eosin, and methylene blue) according to the manufacturer’s protocol (Sigma-Aldrich, St. Louis, MO, USA). Nucleated blood cells were examined under a light microscope at ×1250 in random order. At least 300 nucleated blood cells in each sample were analyzed for cell types. Erythroblasts were detected morphologically [14].

### 2.6. Microspectrophotometry

Microspectrophotometry was performed on an SMP-05 Opton scanning microspectrophotometer to measure the spectra of hemoglobin (Hb) in unstained erythrocytes. The spectrophotometric measurement was only represented on individual erythrocytes, with the extracellular area as a standard reference. The microspectrophotometric method was chosen because of its ability to measure small spectral changes in a limited number of erythrocytes [15]. Cytometric studies were also carried out.

### 2.7. Statistical Analysis

Data analysis was performed with SPSS-19 software. The measurement data were generally non-normally distributed, so the non-parametric Mann–Whitney u-test was used. Initially, the severity of the disease was recorded on a scale of 1–4 scale: 1, normal group; 2, erythroblastosis; 3, low oxygen saturation group; 4, severe disease group. The following erythroblastosis scale was used to record the amount of erythroblasts in the blood: (1) none, (2) 0.1%, (3) 0.3–0.5%, (4) 0.6%, and more. The Spearman correlation coefficient was used for correlation analysis based on the data.

## 3. Results

### 3.1. Basic Characteristics of the Patients

The median age of the 74 patients was 59 years (31–83 years), and 38 (51.3%) were male. There were twelve cases in the low saturation group, nine cases in the group with marked/notable erythroblastosis (at least 1% of erythroblasts in the total nucleated cell count), thirty-five cases in the normal group, and eighteen cases in the severe group with critical form of the disease. The groups did not differ significantly in terms of sex ratio or mean age. The main clinical symptoms of all patients were cough, fever, and fatigue, although patients with critical and severe disease were more likely to have shortness of breath.

### 3.2. Laboratory Results

Owing to the limited quantity of critically sick patient cases, statistical variation was minimized by combining the severe and critically ill groups and comparing them with the regular group.

Ferritin levels were greater in patients with erythroblastosis (ferritin-87.6–346 ng/mL) and severe and critical cases (ferritin-104.8–2929 ng/mL) than in low saturation cases (ferritin-59.8–894 ng/mL). Similar findings were observed in the investigation of CRP levels in serum (Table 1).

There were no statistically significant changes in the peripheral blood cell population composition in any of the groups, as Table 2 demonstrates. An exception is the finding of erythroblasts in the group with severe cases compared to the regular group.

### 3.3. Routine Blood Test and Erythroblastosis

Routine blood tests were performed on all patients. The results showed that the white blood cells (WBC) of the COVID-19 patients were essentially within the normal reference range. A small percentage of individuals (7–8%) had lymphopenia. The consistent finding of erythroblasts in the peripheral blood was the primary feature that distinguished patients with coronavirus infection from the general population. Acidophilic erythroblasts were found in every group of patients in the hospital. In the group with severe coronavirus infection, the percentage of patients with a diagnosis of erythroblastosis ranged from 44% to 5–10% (Table 2). A common finding in SARS-CoV-2 patients (about 30%) was the presence of erythroblasts. It was significantly higher than in the normal group (*p* < 0.01) and more frequent, especially in the severe group.

Patients in the severe group had erythroblasts representing 0.1% to 0.5% of the total nucleated cell population. In addition, erythroblasts that were both polychromatic and basophilic were consistently seen in blood smears from patients with severe disease.

**Figure 1:**
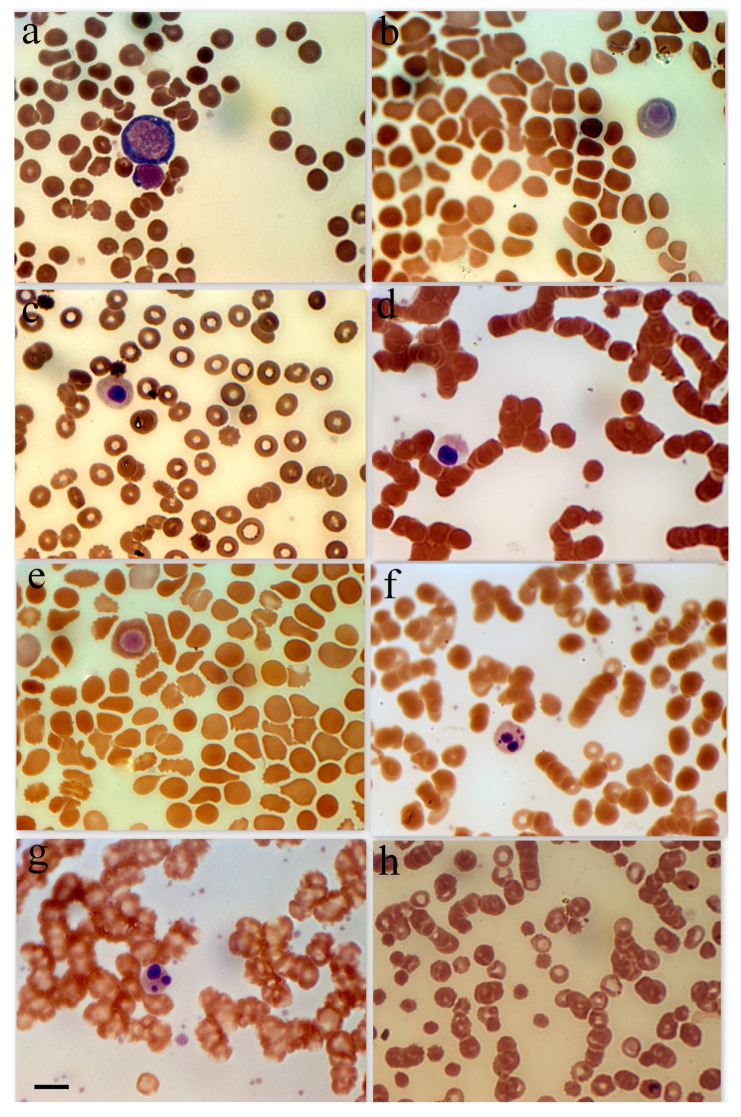
Erythroid cell morphology in peripheral blood in patients with SARS-CoV-2. (**a**) Basophilic erythroblast in a female patient with a severe form of coronavirus. (**b**) Polychromatophilic erythroblast female patient with a moderate form of coronavirus. (**c**) Polychromatophilic erythroblast, female 48-year-old patient with diabetes and hypertension. (**d**) Acidophilic erythroblast, female patient with a severe form of coronavirus. (**e**) Acidophilic erythroblast, female patient with a moderate form of coronavirus. (**f**) Acidophilic erythroblast with a cleaved nucleus, male patient with a moderate form of coronavirus. (**g**) Acidophilic erythroblast with a cleaved nucleus, female patient with a moderate form of coronavirus. (**h**) Anisocytosis in female patients with a severe form of coronavirus. Scale bar 10 µm.

**Figure 2:**
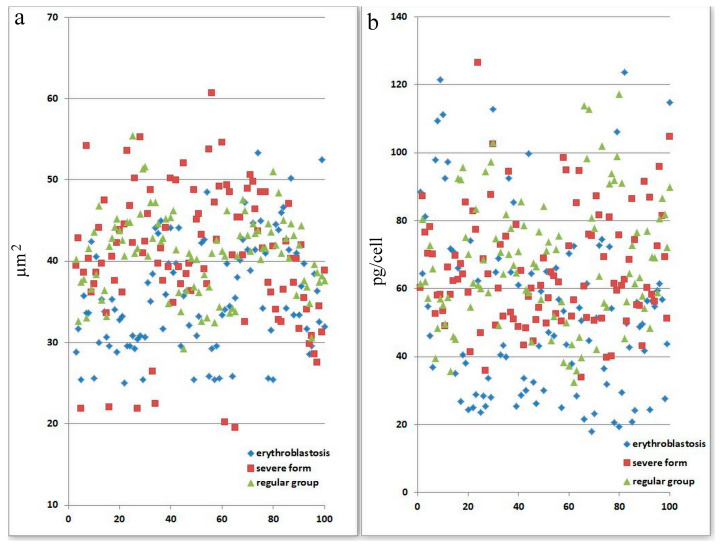
Morphological abnormalities of erythrocytes in patients with SARS-CoV-2 infection. Each point is the average of erythrocytes from three patients, systematized by size. (**a**) Size of erythrocytes. Significant by u-test in the severe group (*p* < 0.05). (**b**) Hb amount in erythrocytes. Significant by u-test in the severe group (*p* < 0.05).

Because of the comparatively high incidence of erythroblastosis in SARS-CoV-2 infection, a separate group of patients (the erythroblastosis group) was established for a more in-depth study of this disease. The group consisted of patients whose erythroblast count was greater than 0.5% of the total nucleated cell population. Anisocytosis and erythroblastosis co-occurred in 16.7% of patients in the severe group (Figure 1h). The degree of anisocytosis in patients with the severe form of SARS-CoV-2 is shown by microcytometry data (Figure 2a). The erythroblastosis group had a lower hemoglobin concentration (Figure 2b).

### 3.4. Influence of Coronavirus Infection on Erythrocyte Parameters, Determined by Microspectrophotometry

Two hundred erythrocytes were used to calculate the mean values of the hemoglobin content, the concentration of a single red blood cell, and the size of the cell. It was discovered that coronavirus infection was the source of variations in Hb adsorption spectra in erythrocytes.

Figure 2 displays information on variations in the adsorption spectra of a single erythrocyte. For panel a, statistical analysis (using the u-test) indicates that there is a significant difference in the severe group (*p* < 0.05), suggesting that erythrocyte size abnormalities are pronounced in patients with severe SARS-CoV-2 infection. For panel b, the severe group shows statistically significant differences in hemoglobin levels per cell (*p* < 0.05). This implies that the severe form of SARS-CoV-2 affects erythrocyte function and hemoglobin content more markedly than the other groups. This figure demonstrates that severe SARS-CoV-2 infection leads to significant morphological and functional abnormalities in erythrocytes, as evidenced by changes in their size and hemoglobin content. These abnormalities might reflect the disease’s systemic impact on oxygen transport and cellular health.

**Figure 3:**
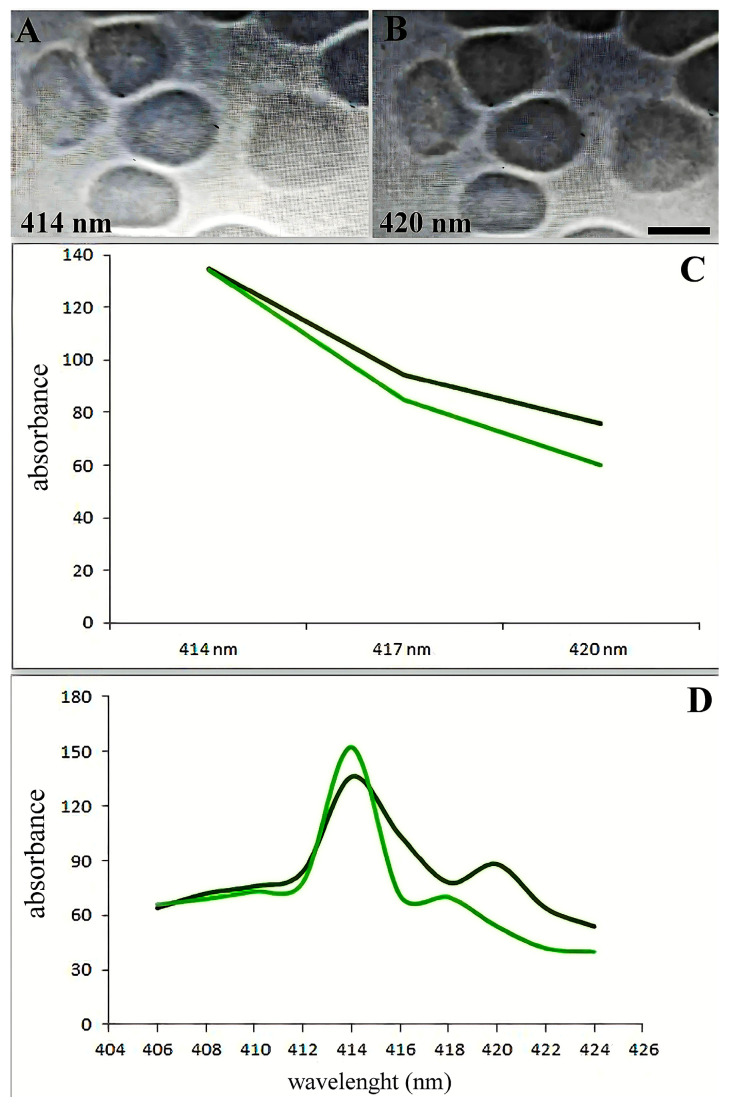
Soret absorption spectroscopy of hemoglobin. The graph shows the microspectrophotometry data of erythrocytes with an identical area (differences less than 1%) on different wavelengths. Black line: main type of erythrocytes. Green line: erythrocytes with variable adsorbance in SARS-CoV-2 infection. (**A**) Erythrocytes in 414 nm wavelengths. (**B**) Erythrocytes in 420 nm wavelengths. Scale bar 5 µm. (**C**) Spectral changes of erythrocytes in patients with SARS-CoV-2 (black curve) and an increase in hemoglobin adsorbance occur about the 420 nm wavelength spectrum. (**D**) Adsorption spectra of single erythrocytes (with the same surface area) were studied by microspectrophotometry on different wavelengths.

The spectra between 414 and 420 nm showed the most alterations (Figure 3A,B). Figure 3C illustrates the variations in adsorption spectra between the primary erythrocyte type and the variable absorption brought on by SARS-CoV-2. The absorption maxima in the 418–422 wavelength range shifted dramatically, as Figure 3D illustrates.

Patients from groups with severe manifestations of the disease and those with erythroblastosis have higher absorption maxima in individual erythrocytes at wavelengths 418–422. According to hemoglobin spectrophotometry, there is a noticeable decrease in hemoglobin absorption as the wavelength increases from 414 to 420 nm. Normally, the erythrocyte population is quite homogeneous (Figure 3D). However, a less pronounced decrease in absorption was observed in some SARS-CoV-2 erythrocytes (20–35% of patients from the severe form of the disease and from the erythroblastosis group; see below) compared to the control (Figure 3D). Patients with erythroblastosis and severe form of SARS-CoV-2 had higher erythrocyte counts and greater Hb absorption at 420 nm according to microspectrophotometry.

**Figure 4:**
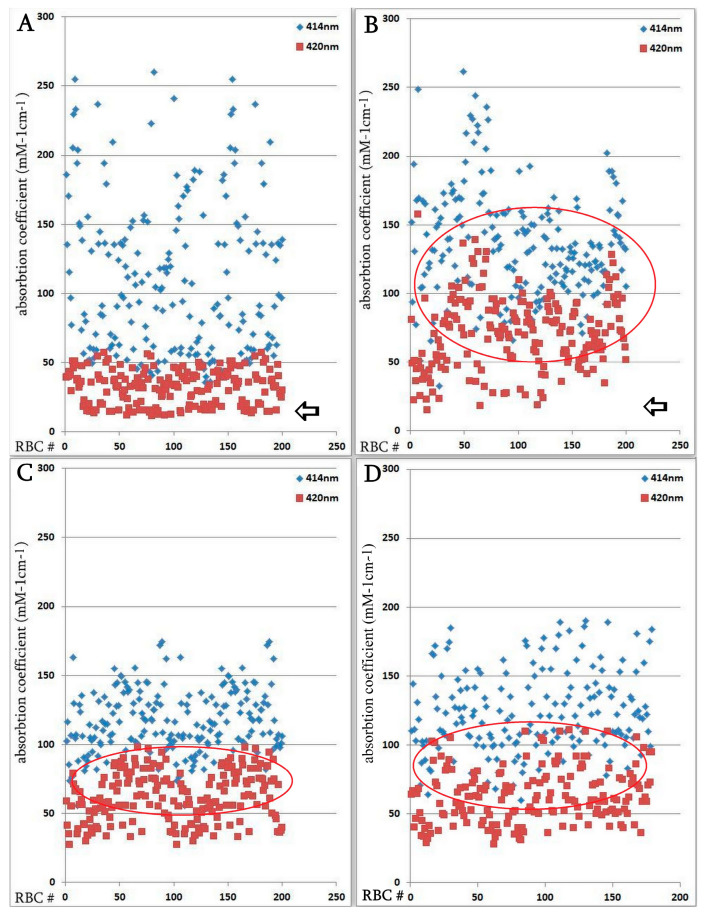
Distribution of single erythrocytes by absorption spectroscopy of hemoglobin on different wavelengths in norm and in patients with SARS-CoV-2. Erythrocytes containing hemoglobin with increased absorption spectra were isolated. Erythrocytes containing hemoglobin with low levels (less than 30 mM 1cm^−1^) of absorption spectra are indicated by arrows. Each point is the average of erythrocytes from three patients, systematized by size. (**A**) Norm. (**B**) Regular group. (**C**) Severe group. Significant by u-test in comparison with norm and regular groups (*p* < 0.05); (**D**) Group with erythroblastosis. Significant by u-test in comparison with norm and regular groups (*p* < 0.05).

The hemoglobin absorption spectra of individual erythrocytes were significantly altered according to microspectrophotometric studies. However, in the severe form of SARS-CoV-2, there was an increase in hemoglobin absorbance within the 420 nm wavelength spectrum (Figure 4); this increase was statistically significant compared with the normal group. Microspectrophotometric studies revealed significant changes in the hemoglobin adsorption spectra of individual erythrocytes in all forms of coronavirus infection compared with healthy individuals (Figure 4A). The appearance of erythrocytes with an increase in hemoglobin absorption spectra at the wavelength of 420 nm was observed in the normal group of patients (Figure 4B), in patients with a severe course of SARS-CoV-2 (Figure 4C) and in the group with erythroblastosis (Figure 4D), this increase was statistically significant (u criterion) compared to healthy individuals. However, in the groups with a severe form of SARS-CoV-2 and with erythroblastosis, erythrocytes containing hemoglobin with low levels (less than 30 mM 1cm^−1^) of absorption spectra (indicated by arrows) disappeared, this increase was statistically significant (u criterion) compared with the usual group and healthy people. The hemoglobin absorption spectra in the erythroblastosis group varied between those of the control group and the severe SARS-CoV-2 disease, but in this group, there are also cells with Hb with increased absorption spectra at 420 nm wavelength. The severe form of SARS-CoV-2 and the control group had different hemoglobin absorbance indices than the erythroblastosis group. Serum ferritin, CRP, and anisocytosis levels are closely related to disease severity, as shown in Table 3.

## 4. Discussion

The changes we found in the peripheral blood after coronavirus-induced pathology resembled the erythropoiesis process. Anisocytosis, variations in Hb absorption spectra, and erythroblast shedding from the bone marrow are the main manifestations of these changes. Most people have moderate or absent coronavirus infections that do not require hospitalization. Hospitalized patients with initially more severe disease have been found to have erythroblastosis. However, contrary to what others have described [3], erythroblastosis was not a common observation in individuals with a worse prognosis. Erythroblasts were found in every group of patients studied. No significant correlation (only trend) (*p* < 0.1) was found between higher levels of erythroblastosis and a worse prognosis or more severe course of the disease. Furthermore, no correlation was observed between the degree of oxygen saturation and elevated erythroblastosis levels, which were equivalent in the normal and severe groups. Hypoxia is often associated with erythroblastosis [16]. Although hemoglobinopathy may play an important role in the overall pathology of SARS-CoV-2, our data did not show a correlation between the amount of erythroblastosis and decreased saturation (only the amount of erythroblastosis tended to correlate with disease severity) [17,18]. Thus, both the direct targeting of erythroid precursors by SARS-CoV-2 and the systemic hyperinflammation that characterizes individuals with severe disease may have an indirect effect on erythropoiesis abnormalities such as erythroblastosis [17,19]. These findings, showing that the virus was still present in erythroid cells 14 days later without affecting their viability, suggest that direct infection may be the cause of the erythroblastosis seen in severely affected individuals.

ACE2, CD147, CD26, and other receptors on erythrocytes and/or blood cell precursors may mediate the effect of the virus on hemoglobin [3]. In this regard, recommendations have been made [2] regarding potential interactions between COVID-19 and hemoglobin, which could decrease the total content and oxygen affinity of hemoglobin. Changes in the UV spectrum of hemoglobin may also be caused by an increase in the metHb fraction in SARS-CoV-2 [20]. However, the literature data suggest that changes in the UV spectra of hemoglobin cannot be fully explained by changes in metHb or oxyHb alone [21,22].

Antimalarial drugs that may also have anti-SARS-CoV-2 activity target Plasmodium, which has a similar route of erythrocyte entry via CD147. They have been used to prevent non-structural SARS-CoV-2 proteins from forming a porphyrin complex and attacking hemoglobin [17].

Ferritin levels increase in patients with the progressive form of SARS-CoV-2 and poor prognosis, according to several articles [19,23,24]. Acute phase reactant serum ferritin levels correlate with the severity of infection-related acute and chronic inflammation [25]. Our results support the findings of the cited authors, who concluded that the group of severe SARS-CoV-2 cases had the highest serum ferritin levels. A significant percentage of the ferritin levels examined were higher in the group of patients with low oxygen saturation than in the group of normal patients. Some SARS-CoV-2 patients had elevated ferritin levels, which may indicate an inflammatory response or be related to viral entry into the bloodstream and its effect on iron metabolism [26]. In addition, we found anisocytosis, which is a marker of anemia. In the general population, anisocytosis is also associated with increased all-cause mortality [27]. According to our findings, elevated ferritin levels were not associated with erythroblastosis but rather with anisocytosis, altered microspectrophotometric characteristics of hemoglobin in erythrocytes, and disease severity. Abnormalities in erythrocyte size distribution have been associated with inflammation in a number of studies [27,28]. Despite a strong correlation between elevated anisocytosis and CRP, this phenomenon is only part of the complicated erythrocyte pathology resulting from SARS-CoV-2 infection. This type of interaction can lead to a decrease in the amount of hemoglobin available to carry oxygen, shift the oxygen dissociation curve, and reduce the affinity of oxygen for hemoglobin.

## 5. Conclusions

Increased levels of ferritin, CRP, anisocytosis, and partially increased Hb absorption at 420 nm may be positively correlated with adverse outcomes in SARS-CoV-2 infection.

## Figures and Tables

**Table 1:** Clinical characteristics of the 74 patients with SARS-CoV-2.

Characteristic	Regular Group (*n* = 35)	Severe Group (*n* = 18)	Low Saturation Group (*n* = 12)	Erythroblastosis Group (*n* = 9)
Age (year)	61 ± 4.5	58 ± 3.3	56 ± 4.8	57 ± 5.5
Male sex (%, N)	51.4 (18)	50.0 (9)	50.0 (6)	55.5 (5)
Symptoms (%, N)				
Fever	80% (28)	94.5% (17)	16.7 (2)	66.7% (6)
Cough	77.1% (27)	22.2% (4)	75% (8)	22.2% (2)
Loss of smell/taste	57.1 (20)	16.7% (3)	16.7% (2)	-
Shortness of breath	22.8% (8)	16.7% (3)	50% (6)	22.2% (2)
O_2_ saturation	92.2 ± 2.8	88.5 ± 4.9	75.7 ± 5.3	91.2 ± 5.1
Anorexia	8.7% (3)	5.5% (1)	-	-
Diarrhea	5.7% (2)	5.5% (1)	-	-
Fatigue	82.9% (29)	22.2% (4)	83.3% (10)	77.8% (7)
Myalgia or arthralgia	28.6% (10)	16.7% (3)	25% (3)	44.4% (4)
Coexisting disorder (%, N)				
Hypertension	42.9% (15)	38.8% (7)	33.3% (4)	44.4% (4)
Diabetes	31.4% (11)	27.8% (5)	25% (3)	22.2% (2)
Coronary heart disease	8.7% (3)	5.5% (1)	8.3% (1)	22.2% (2)
Cerebrovascular disease	-	5.5% (1)	-	-
Chronic renal disease	2.9% (1)	5.5% (1)	-	-
Malignant tumor	5.7% (2)	5.5% (1)	-	-
other coexisting chronic disorder	20% (7)	11.1% (2)	8.3% (1)	-
Ferritin level (ng/mL)	87.6–346	104.8–2929 *	59.8–894	87–285
CRP (mg/L)	46.5 (8–81)	89.3 (21–231)	47.9 (14–95)	19.1 (7–72)

* Significant compared to Regular group and group with Erythroblastosis (*p* < 0.05), tendency compared to Low Saturation Group (*p* < 0.1).

**Table 2:** Blood cell populations.

Blood Cells	Regular Group (*n* = 35) *	Severe Group (*n* = 18) **	Low Saturation Group (*n* = 12) ***	Erythroblastosis Group (*n* = 9)
Basophilic erythroblast	-	0.1	-	0.7
Polychromatophilic erythroblast	-	0.1	-	0.7
Acidophilic erythroblast	0.1	0.3	0.1	0.1
Lymphoblast	1.8 ± 1.4	2.5	1.4	2.1
Lymphocyte	22.0 ± 6.4	25.3	28.8	28.5
Lymphocyte aberrant	1.1 ± 2.1	0.4	1.4	1.8
Monoblast	0.4 ± 0.7	0.3	0.4	0.7
Monocyte	2.1 ± 1.4	2.6	2.6	1.6
Myeloid cell	2.8 ± 2.5	1.6	3.7	1.3
Metamyelocyte	14.7 ± 7.4	16.3	17.7	12.2
Band neutrophil	39.9 ± 9.6	36.8	34.4	39.1
Segmented neutrophil	12.3 ± 1.1	10.9	7.7	8.2
Pathological neutrophil	0.8 ± 0.9	0.7	0.7	1.6
Eosinophil	0.4 ± 0.2	0.9	0.2	0.5
Basophil	0.1 ± 0.2	0.1	0.0	0.1
Destructed cells	1.3 ± 1.2	1.1	1.0	0.9

* Erythroblastosis present in 5.7% of cases (2 patients from 35). ** Erythroblastosis present in 44.4% of cases (8 patients from 18). *** Saturation below 85%.

**Table 3:** Correlation analysis of red blood alterations and disease severity by Spearman coefficient.

	Disease Severity	Erythroblastosis	Ferritin	CRP	Increased Hb Absorption on 420 nm (%)	Anisocytosis (%)	Decreased Hb in Erythrocyte (%)
disease severity	1.000	0.316	1.000 *	1.000 *	0.800	1.000 *	−0.105
Erythroblastosis	0.316	1.000	0.316	0.316	0.632	0.316	0.833
ferritin levels in serum	1.000 *	0.316	1.000	1.000 *	0.800 **	1.000 *	−0.105
CRP levels in serum	1.000 *	0.316	1.000 *	1.000	0.800 **	1.000 *	−0.105
increase in Hb absorption on 420 nm (%)	0.800 **	0.632	0.800 **	0.800 **	1.000	0.800 **	0.105
anisocytosis (%)	1.000 *	0.316	1.000 *	1.000 *	0.800 **	1.000	−0.105
decreased Hb amount in erythrocyte (%)	−0.105	0.833	−0.105	−0.105	0.105	−0.105	1.000

* Correlation is significant at the 0.01 level (2-tailed). ** Tendency at 0.1 level (2-tailed).

## Data Availability

The original contributions presented in the study are included in the article, further inquiries can be directed to the corresponding author.
